# Myelination of parvalbumin interneurons: a parsimonious locus of pathophysiological convergence in schizophrenia

**DOI:** 10.1038/mp.2016.147

**Published:** 2016-09-20

**Authors:** J Stedehouder, S A Kushner

**Affiliations:** 1 Department of Psychiatry, Erasmus University Medical Center, Rotterdam, The Netherlands

## Abstract

Schizophrenia is a debilitating psychiatric disorder characterized by positive, negative and cognitive symptoms. Despite more than a century of research, the neurobiological mechanism underlying schizophrenia remains elusive. White matter abnormalities and interneuron dysfunction are the most widely replicated cellular neuropathological alterations in patients with schizophrenia. However, a unifying model incorporating these findings has not yet been established. Here, we propose that myelination of fast-spiking parvalbumin (PV) interneurons could be an important locus of pathophysiological convergence in schizophrenia. Myelination of interneurons has been demonstrated across a wide diversity of brain regions and appears highly specific for the PV interneuron subclass. Given the critical influence of fast-spiking PV interneurons for mediating oscillations in the gamma frequency range (~30–120 Hz), PV myelination is well positioned to optimize action potential fidelity and metabolic homeostasis. We discuss this hypothesis with consideration of data from human postmortem studies, *in vivo* brain imaging and electrophysiology, and molecular genetics, as well as fundamental and translational studies in rodent models. Together, the parvalbumin interneuron myelination hypothesis provides a falsifiable model for guiding future studies of schizophrenia pathophysiology.

Schizophrenia is a chronically debilitating psychiatric disorder with a lifetime prevalence of ~1%.^1^ Patients with schizophrenia classically exhibit a constellation of positive, negative and cognitive symptoms.^2^ Although many theories have been proposed, the precise neurobiological mechanism underlying schizophrenia has remained elusive. The most widely described models have been the dopamine^3^ and glutamate hypotheses,[Bibr bib4]^4^ although in recent years models regarding interneuron dysfunction^5^ and myelination abnormalities^6^ have gained increasing support.

In this Perspective, we hypothesize that previous observations of interneuron dysfunction and myelination abnormalities in schizophrenia might converge on the altered myelination of fast-spiking parvalbumin (PV) interneurons. First, we summarize the major evidence supporting interneuron dysfunction and myelination abnormalities in schizophrenia. Next, we summarize electron microscopy and immunofluorescence studies that convincingly demonstrate interneuron myelination, which frequently occurs on fast-spiking PV interneurons. Finally, we discuss how impairments in myelination of PV interneurons could lead to consequent abnormalities in gamma synchronization and ultimately give rise to the symptoms which define schizophrenia.

## Parvalbumin interneuron dysfunction in schizophrenia

Deficits in GABAergic signaling have been widely proposed as a fundamental pathophysiological mechanism underlying schizophrenia.^7^ More specifically, several recent lines of evidence including human postmortem studies, genetics and *in vivo* electrophysiological recordings in patients and translational mouse models have identified fast-spiking PV interneurons as the major GABAergic cell-type affected in schizophrenia (Table 1).

Expression of GAD67—the predominant gamma-aminobutyric acid (GABA) synthesizing enzyme—has consistently been found to be reduced at both the messenger RNA and protein levels in several brain regions of patients with schizophrenia, a finding that has been well controlled for confounding factors.^8, 9, 10, 11, 12, 13, 14^ Downregulation of GAD67 messenger RNA has been reported in ~30% of dorsolateral prefrontal cortex interneurons^15, 16^ and entirely undetectable in ~50% of PV interneurons.^17^ Expression of PV messenger RNA^18, 19, 20^ and protein^21^ is also reduced in schizophrenia, while the neuronal density of cortical PV interneurons is unchanged^22, 23, 24, 25^ (but see also ref. 52). Since the expression of both PV and GAD67 are experience-dependent^26^—and GAD67 and PV expression are highly correlated^26^—their shared downregulation suggests a functional impairment of fast-spiking interneurons.^27^ Morphologically, PV cell inputs onto pyramidal neurons have no discernible alterations^21^ suggesting a primary functional abnormality of PV interneurons. Consistent with these neuropathological findings, *in vivo* positron emission tomography (PET) imaging has demonstrated widespread alterations of cortical GABA transmission in schizophrenia, a finding that was most prominent in the subset of patients who were antipsychotic-naïve.^28^ Altogether, these results provide compelling evidence of cortical PV interneuron dysfunction in schizophrenia.

PV interneurons are essential in generating cortical oscillations in the gamma range (~30–120 Hz), mediated by synchronized inhibition of large pyramidal cell ensembles.^29, 30^ Through rhythmic perisomatic inhibition onto surrounding pyramidal cells, synchronous ensembles of PV cells evoke high-frequency gamma oscillations in the cerebral cortex.^31, 32, 33^ Gamma synchrony has been shown to function critically across a range of cognitive functions, including working memory and attention^34^ with well-replicated abnormalities in schizophrenia.^5, 35^ Abnormalities in other frequency bands such as theta and alpha have also been reported in schizophrenia, but the neural mechanisms underlying these frequencies remain less well understood.^35^

Electroencephalographic studies in schizophrenia have shown a reduced amplitude and impaired phase locking of gamma band activity over frontal areas while assessing working memory and executive functioning tasks.^35^ Although some studies have observed concurrent increases in gamma band activity at rest, this finding has been less well replicated.^35^ Taken together, impairments of *in vivo* gamma oscillations in patients with schizophrenia are highly consistent with the PV interneuron abnormalities observed by postmortem histopathology.

The classical onset of schizophrenia occurs within a relatively narrow window of neurodevelopment, between ~18 and 25 years of age.^2^ This late adolescent age of onset has often been attributed to the ongoing functional maturation of the brain during this neurodevelopmental critical period.^2^ Specifically in late adolescence, rates of synaptic pruning and myelination become asymptotic for which impairments in these processes have been linked to the disease onset.^2^ Notably, maturation of gamma band synchrony also occurs during late adolescence^35^ which coincides developmentally with the clinical onset of schizophrenia.^1^

In addition to *in vivo* brain imaging, electroencephalographic recordings and postmortem histopathology, molecular genetic studies of schizophrenia have also revealed an important contribution of interneuron dysfunction to the pathophysiology of schizophrenia. A recent genetic study of copy number variation has now provided causal evidence for GABAergic dysfunction in the etiology of schizophrenia.^36^ In this study, Pocklington *et al.* performed a functional gene set analysis for enriched biological mechanisms using a large schizophrenia case-control dataset and found that copy number variations were significantly enriched in cases for genes responsible for inhibitory neurotransmission (in particular the GABA_A_ receptor complex), glutamatergic neurotransmission, long-term synaptic plasticity and associative learning. The genetic variant with the highest known risk for schizophrenia is the 22q11 microdeletion which has a penetrance of ~40%.^37, 38^ Transgenic mouse models have been generated to investigate the underlying neurobiology conferred by 22q11 microdeletion. *Df(16)A* mice harboring a 27-gene microdeletion syntenic to a 1.5 Mb region of human 22q11.2 exhibit similar brain abnormalities as found in human 22q11 microdeletion carriers, including cortico-cerebellar, cortico-striatal and cortico-limbic circuits.^39^ Moreover, multiple different mouse models of 22q11 microdeletion have replicated a cell-type specific impairment in PV interneurons and disrupted local synchrony of neural activity, consistent with the deficit in gamma oscillations observed in schizophrenia.^40, 41, 42^

Evidence for interneuron dysfunction in schizophrenia has also been supported by a wide variety of non-genetic rodent models.^43^ The major examples include pharmacological NMDA receptor antagonism and neurodevelopmental immunological challenge, both of which consistently exhibit synaptic and network abnormalities reminiscent of schizophrenia pathophysiology. Specifically, these studies have identified electrophysiological changes in local microcircuit connectivity and synaptic plasticity, with alterations in excitation/inhibition balance and gamma band synchronization.

Taken together, the combination of genetic, postmortem, and *in vivo* electrophysiological and functional imaging results from human clinical studies of schizophrenia converge with translational rodent modeling to identify fast-spiking PV interneuron dysfunction as a major pathophysiological mechanism underlying schizophrenia etiology.

## Myelination abnormalities in schizophrenia

Independent of PV interneuron alterations, myelination abnormalities have also been extensively implicated in schizophrenia through both *in vivo* brain imaging and postmortem assessments (Table 1). Numerous diffusion tensor imaging studies have been published for schizophrenia (reviewed in ref. 6), of which the overwhelming consensus has been the association of schizophrenia with globally decreased fractional anisotropy. Notably, the decrease in fractional anisotropy appears to become more severe with increasing age and illness duration.^44^ Many of the early brain imaging studies of schizophrenia were performed in cohorts with extensive histories of psychotropic medication, inpatient hospitalization, smoking and medical co-morbidities, which could have a confounding deleterious influence on white matter integrity. Thus, an important question has been whether myelination abnormalities are already present in drug-naïve patients with first-episode schizophrenia who have never received psychotropic medication. Recently, several diffusion tensor imaging studies have been performed in such cohorts,^44, 45, 46, 47, 48, 49, 50, 51, 52, 53, 54, 55, 56, 57, 58^ holding the potential to directly evaluate these potential confounders. Indeed, across a range of different methodologies, studies of drug-naïve first-episode schizophrenia have consistently demonstrated similar, albeit less severe, myelination abnormalities as observed in chronic illness. Importantly, these studies confirm that a global impairment of myelin integrity is already present at the time of the initial clinical onset of psychotic symptoms in schizophrenia. Accordingly, these findings support a model by which myelination abnormalities function critically in the pathophysiology of schizophrenia.

The late adolescent age of onset for schizophrenia closely overlaps with the maturation of prefrontal cortex myelination.^59^ The time course of myelination in humans has been elegantly detailed through longitudinal *in vivo* imaging and postmortem cross-sectional studies demonstrating rapid early postnatal white matter development in the first 12 months^60^ followed by a slower but steady increase until late adolescence.^61, 62^ Comparative mammalian evolutionary studies have demonstrated that humans exhibit a particularly extended neurodevelopmental time course of neocortical myelination.^63^ Although myelination in humans peaks in late adolescence, for non-human primates and rodents the peak of myelination occurs significantly earlier in development.^63^ Together, the current best evidence demonstrates that the onset of schizophrenia closely coincides with the peak of myelination in human brain development.

In addition to the well-replicated finding of *in vivo* white matter abnormalities in schizophrenia, postmortem gene expression analyses have also identified alterations in myelination regulatory pathways. Several studies have reported a broad reduction in the expression of genes with demonstrated function in the oligodendrocyte lineage.^64, 65, 66, 67, 68, 69, 70, 71^ Using microarray-based transcriptome analysis with quantitative PCR validation, abnormalities in oligodendrocyte lineage genes have been found in both frontal white and gray matter,^64, 65, 68^ subcortical regions,^66, 69^ occipital cortex^70^ and temporal cortex.^71^ The alignment between *in vivo* brain imaging findings and postmortem gene expression analyses is highly consistent with a central importance of myelination abnormalities in schizophrenia pathophysiology. Notably, many of the same oligodendrocyte and myelination genes found to be altered in schizophrenia also exhibit consistent increases during normal brain development precisely during adolescence,^72^ again consistent with the association between the late adolescent age of onset in schizophrenia and the peak of myelination.

Compared with the abundance of brain imaging and gene expression studies of myelination and oligodendrocytes, postmortem stereological analysis of oligodendrocyte lineage cell types are scarce. From the few studies that have been performed, stereological quantification of myelinating oligodendrocytes have revealed widespread reductions in schizophrenia (Table 1).^73, 74, 75, 76, 77, 78, 79, 80, 81, 82, 83^ Reductions in oligodendrocyte numbers have been shown in the white and gray matter of BA9,^73, 74, 75, 76, 83^ white and gray matter of BA10,^77, 78^ posterior hippocampal subregion CA4,^79^ internal capsule,^80^ nucleus basalis^81^ and anterior thalamic nucleus.^82^ In contrast, oligodendrocyte numbers appear unchanged within the substantia nigra,^84^ callosal genu^85^ and subgenual cingulum.^85^ Furthermore, one study failed to find differences in oligodendrocyte number within any subregion of the hippocampus.^86^ In addition, a few studies have reported seemingly paradoxical increases in the number of myelinating oligodendrocytes in frontal white matter^87^ and basolateral amygdala.^88^ Although caution is warranted given the limited number of studies and differences in methodology, the emerging picture is one of small but consistent reductions of myelinating oligodendrocytes in schizophrenia (~14% reduction^73, 74, 75, 76, 77, 78, 79, 80, 81, 82^). However, an important unanswered question is whether the observed reduction of myelinating oligodendrocytes is cell-type specific or also extends to other less differentiated cell types within the oligodendrocyte lineage.

A recent study is the first to report a stereological analysis of oligodendrocyte precursors cells (OPCs),^83^ also known as neuron-glial antigen 2 (NG2) cells due to their abundant expression of the NG2 protein. The number of frontal white matter OPCs were unchanged while the total population of oligodendrocyte lineage cells was reduced, thereby suggesting that the reduction in oligodendrocyte lineage cells occurs predominantly in more differentiated cell types. Furthermore, oligodendrocyte lineage-specific transcriptome analysis and immunohistochemical labeling independently suggested an impairment of OPC differentiation towards mature oligodendrocytes. Given that OPCs are the exclusive progenitor cell population of myelinating oligodendrocytes, more knowledge of the regulation and function of OPCs in schizophrenia would better clarify whether the observed reductions in myelinating oligodendrocytes are a consequence of abnormalities that have occurred upstream in the myelination lineage or the consequence of a downstream cell-type specific loss of myelinating oligodendrocytes.

Two studies have examined myelination at the ultrastructural level in schizophrenia. The major findings involved myelinated axons and oligodendrocytes, in frontal cortex white and gray matter.^89, 90^ The observed pathological features included alterations in the morphology of the myelin sheath and the frequency of axonal degeneration within morphologically intact myelin segments. Notably, the effect sizes were larger in gray matter compared with white matter regions.^89, 90^

With regard to *in vivo* and postmortem findings, the possibility remains that the observed myelination abnormalities in schizophrenia could result from primary and/or secondary disturbances of neuronal signaling.^91^ Therefore, genetic studies provide a unique opportunity to investigate etiological mechanisms of schizophrenia while avoiding the potential confounds of antipsychotic medication and secondary disease effects. Notably, recent studies have shown using genome-wide association study (GWAS) data that myelination/oligodendrocyte gene sets are significantly associated with both the risk of schizophrenia^92, 93, 94^ and the severity of deficits in white matter integrity.^95^ Moreover, the most recent GWAS results for schizophrenia exhibited a significant enrichment of genes expressed in mature oligodendrocytes,^96^ together suggesting a convergence of common variant risk on myelination.

Although important questions remain unanswered, GWAS results implicating myelination as an etiological mechanism, *in vivo* imaging demonstrating well-replicated myelination abnormalities, human postmortem histopathology showing replicated decreases in the number and ultrastructure of oligodendrocytes, and gene profiling studies demonstrating replicated changes in oligodendrocyte expression, together provide compelling support for myelination as a major pathophysiological mechanism of schizophrenia.

## Myelination of parvalbumin interneurons

An increasing number of studies has revealed the unexpectedly extensive myelination of GABAergic interneurons (Table 2), predominantly fast-spiking PV basket cells (Table 3), in cortical gray matter and other regions throughout the brain.^97, 98, 99, 100, 101, 102, 103, 104, 105, 106, 107, 108, 109, 110, 111, 112, 113, 114, 115, 116, 117, 118, 119, 120, 121, 122, 123^ Myelination of cortical GABAergic basket cells was first reported over 30 years ago in the cat visual cortex by electron microscopy.^98, 99, 100^ In non-human primates, GABAergic axons in layers III–V are myelinated in sensorimotor^106, 107, 108^ and temporal^102^ cortices, a finding that had already been hinted at several years earlier.^105^ Myelinated GABAergic interneurons were subsequently identified in the cat superior colliculus,^118^ as well as in the red nucleus^110^ and hypoglossal nucleus^109^ of the monkey. Although the relative distribution of myelination across interneuron subtypes has not yet been quantitatively determined, a consistent qualitative observation has been that a high proportion of the GABA-labeled terminals of myelinated axons exhibit localized somatic targeting suggestive of basket cells.^106^ Moreover, direct ultrastructural evidence for basket cell myelination has also been demonstrated in visual cortex of cat^98, 99^ and rat.^112^

Basket cells have been reported to be myelinated in occipital^112^ and somatosensory^114^ cortex of the rat. PV-immunoreactive myelinated neurons have been identified in the rat entorhinal cortex,^119^ hippocampus^122^ and striatum.^123^ In the rat entorhinal cortex, myelinated PV axons were found extensively across all cortical layers, interspersed with unmyelinated axonal segments.^119^ Furthermore, myelinated GABAergic neurons have been identified in the rodent hippocampus,^111, 117^ thalamus^113, 120^ and inferior colliculus,^121^ although these studies were performed largely without interneuron subtype-specific labeling. However in one notable exception, myelinated rat hippocampal GABAergic neurons were confirmed as PV interneurons.^117^ Moreover, the vast majority of septohippocampal PV, but not cholinergic, fibers are myelinated.^115, 116, 124^

Recently, a combinatorial study using array tomography and fluorescence microscopy found a large abundance of myelinated PV axons in adult mouse somatosensory cortex.^125^ Here, the authors found that 25–50% of all cortical myelinated axons are GABAergic, and that nearly all of these are PV-expressing interneurons, a finding that has been independently observed in the adolescent mouse visual cortex.^97^ Notably, although myelin thickness was similar between GABAergic and non-GABAergic axons, myelinated GABAergic axons had a higher average g-ratio (ratio between the inner axonal diameter and the outer diameter of the myelin sheath), shorter internode length, and shorter nodes of Ranvier than non-GABAergic axons.^125^

Few studies have reported attempts to examine myelination of interneurons in human cortex. Myelination of PV cells in the human hippocampus^103^ and claustrum^104^ has been confirmed by electron microscopy. Furthermore, myelinated GABAergic^102^ interneurons have been incidentally observed in the human frontal cortex, including with PV subtype specification.^101^ Thus, although sparsely documented, PV interneuron myelination appears to be widespread throughout the brain and evolutionarily conserved among mammals. More detailed and comprehensive studies are required to quantify the relative proportion of myelinated PV interneurons, their developmental time course of myelination compared to pyramidal neurons, subcellular distribution of segmental myelination, and brain region distribution, as well as the functional neurophysiological implications of interneuron myelination.

Notably, we have not found any report demonstrating myelination of cortical somatostatin or neuropeptide Y interneurons, despite numerous electron microscopic studies in a variety of mammalian species,^126, 127, 128, 129^ thereby suggesting a high specificity for the PV subclass of GABAergic interneurons. In contrast, non-PV interneuron myelination has been sporadically reported in subcortical regions, for example, in sparse small-diameter axons of the rat internal capsule^130^ and in the cat claustrum.^131^ This suggests that at least within the cerebral cortex, PV cells are the predominant myelinated interneuron subtype while in subcortical brain regions the cell-type distribution of myelinated interneurons may be less strict.

Recently, it has been shown that PV interneurons establish direct functional soma-targeted contacts with OPCs in cortical layer V.^132^ Synaptic input from local GABAergic interneurons has been shown to dynamically regulate OPC differentiation to oligodendrocytes.^133^ OPCs receive strong GABAergic synaptic input from PV, and to a lesser extent from non-PV, interneurons.^132^ Notably, the peak neurodevelopmental period of interneuron-OPC connectivity (P10-P14) would thus position interneuron myelination precisely in the window following the initial onset of GABAergic burst firing, but before maturation of high-frequency gamma oscillations.^134^ This also closely aligns with the timing of human frontal cortex oligodendrocyte development which plateaus in early adulthood and is highly distinct from white matter development in which oligodendrocytes have already reached their maximum number by ~5 years of age.^135^ Moreover, in further contrast to white matter, frontal cortex gray matter exhibits a substantial turnover of oligodendrocytes and myelin that persists throughout adulthood.^135^ Analogously, rodent studies have demonstrated that OPCs exhibit important distinctions in their physiology, proliferation and differentiation between gray and white matter in rodents.^136^ Therefore, regional differences in human OPCs are also not unlikely.

Interestingly, direct contacts of interneurons onto OPCs^137^ are only locally distributed, reaching a typical maximum distance of 50–70 μm,^132^ which is highly similar to the estimate for the maximal length of OPC processes. An interesting question remains why interneurons have such a restricted spatial localization of their connectivity onto OPCs, since PV cells establish synaptic contacts with pyramidal cells across a distance approximately six times larger.^138^ One possibility is that OPCs utilize reciprocal synaptic input to regulate their proliferative drive. Alternatively, it may be that myelination preferentially occurs on proximal axonal segments, in close apposition to the observed localization of OPCs and allowing for rapid differentiation to oligodendrocytes with enhanced myelination plasticity.

## Potential functions of interneuron myelination

PV interneurons function to synchronize pyramidal cell ensembles, and thereby generate high-frequency oscillations.^139^ Since cortical PV axonal arborization is widely ramified and distributed over distances of up to 300 μm,^138^ there might be considerable benefits of myelination for optimizing the fidelity of fast action potential transmission. Indeed, computational modeling has suggested a unique contribution of (interneuron) conductance delays in the dynamics of gamma frequency oscillations.^140^ Evidence exists that nodes of Ranvier begin forming before the onset of myelination,^117^ a mechanism specific for GABAergic neurons, which enhances axonal conduction of action potentials without myelin. Thus, in addition to simply increasing the speed of action potential propagation, myelin could function to ensure the integrity of precisely timed action potentials, as has been proposed for myelinated excitatory axons.^141^ Myelin plasticity would then have the potential to support the local synchronization of action potentials necessary for generating high-frequency oscillations.^142^ Indeed, myelinated axons exhibit both higher conduction velocities and enhanced long-range coherence.^143^ Although non-PV cortical interneuron subtypes (e.g., somatostatin, VIP) exhibit synaptic connectivity across similar distances,^138^ their lack of influence in maintaining high-frequency oscillations is consistent with their absence of myelination. Furthermore, the activity-dependence of myelination^144^ might permit dynamically regulated influences on the fidelity of fast action potential transmission and high-frequency oscillations.

Furthermore, myelin could provide metabolic and trophic support for energetically costly PV cells. PV cell characteristics, including high-frequency spiking and rapid action potential kinetics, require a particularly high energy utilization through predominantly mitochondrial oxidative phosphorylation.^145^ Gamma band synchrony, closely linked to cognition, is highly sensitive to metabolic disruption. Furthermore, compared with pyramidal cells, PV cells exhibit high densities of mitochondria and expression of cytochrome *c* and cytochrome *c* oxidase, proteins crucial for the electron transport chain. Moreover, PV cell-specific disruption of cytochrome oxidase assembly leads to changes in PV cell intrinsic excitability, afferent synaptic input, and gamma/theta oscillations, as well as schizophrenia-related behavioral impairments in sensory gating and social behavior.^146^

During gamma oscillations, peak oxygen consumption approaches the demand observed during seizures and mitochondrial oxidative capacity operates near its functional limit.^145^ Metabolic and trophic support conferred by myelination^147, 148^ might therefore allow PV axons to optimize their energy utilization. Consistent with the importance of myelination in regulating axonal energy metabolism is the considerable discrepancy of mitochondria content (30-fold) in myelinated versus unmyelinated axons.^149^ Myelin has been proposed to regulate axonal energy metabolism via the monocarboxylate transporter 1 channel.^147, 148^ Furthermore, the high-peak oxygen consumption of PV cells during gamma band synchrony could require the additional lactate provided by oligodendrocytes.

Taken together, the electrophysiological dynamics of fast-spiking PV interneurons, their dense branching onto pyramidal neurons requiring finely tuned temporally synchronized inhibition, and their high-energy consumption are likely interdependent mechanisms governed by PV interneuron myelination.

## Implications for schizophrenia

Both interneuron dysfunction and myelination abnormalities have been independently proposed as important contributors to the underlying pathophysiology of schizophrenia. These mechanisms have each amassed convincing support from postmortem histopathology, *in vivo* imaging and electrophysiology, genetics and neurodevelopment (Table 1). However, neither hypothesis is capable of accounting for the full set of clinical research findings in schizophrenia. In contrast, interneuron myelination brings together both of these models, explains a more comprehensive portion of the existing data, and offers a well-defined falsifiable model.

Impairments of PV interneuron myelination could directly contribute to schizophrenia through several mechanisms. Impaired action potential fidelity, energy restrictions during highly-demanding cognitive tasks, aberrant axonal branching and a higher occurrence of ectopic action potentials could each independently, or in combination, disrupt inhibitory network function. Such changes to PV interneurons would likely result in abnormalities of local gamma synchronization, with a potential further impact on the integrity of long-range thalamocortical and cortico-striatal circuits, and striatal dopamine signaling, ultimately giving rise to schizophrenia symptoms.

In this Perspective, we have proposed the novel hypothesis that altered myelination of PV interneurons might function prominently in the pathophysiology of schizophrenia. However, many questions remain to be answered. At what point during development does interneuron myelination occur and to what extent does this coincide with the clinical symptoms of schizophrenia? Does interneuron myelination vary across brain regions? Is cortical interneuron myelination truly reserved for fast-spiking PV interneurons, or are non-fast-spiking interneurons (e.g., somatostatin, VIP) myelinated as well? How does the plasticity of PV interneuron myelination compare with that of glutamatergic axons? And perhaps most importantly, to what extent might PV interneuron myelination represent an etiological pathophysiology and therapeutic target for schizophrenia?

Future studies to examine the parvalbumin interneuron myelination hypothesis could be approached through a variety of methods. In particular, the most important experiments would include: (a) detailed histological assessments of subtype-specific interneuron axonal myelination in postmortem brain tissue from patients with schizophrenia, (b) corresponding functional studies in rodent models of schizophrenia to directly assess the causality of alterations in parvalbumin interneuron myelination on behavioral and electrophysiological phenotypes, (c) electrophysiological studies of rodent models with temporally-controlled and cell-type specific disruption of myelination and (d) functional genomic studies on the effect of schizophrenia risk variants on (interneuron) myelination, for example, by utilizing human induced pluripotent stem cells or genetically modified mice.

## Figures and Tables

**Table 1:** Comparison of interneuron and myelination data for schizophrenia

	*Interneuron dysfunction*	*Myelination abnormalities*
Schizophrenia age of onset	Maturation of PV cells^2^ Emergence of high-frequency oscillations^35^	Peak of myelination^63^ Development of frontal gray matter oligodendrocytes^135^
Postmortem findings	PV mRNA and protein decreased^18, 19, 20, 21^ GAD67 mRNA and protein decreased^8, 9, 10, 11, 12, 13, 14^ Transcriptional changes in PV cells^150^	Abnormal myelin/oligodendrocyte gene expression^64, 65, 66, 67, 68, 69, 70, 71^ Lower oligodendrocyte numbers^73, 74, 75, 76, 77, 78, 79, 80, 81, 82, 83^ Ultrastructural abnormalities^89, 90^ Transcriptional changes in oligodendrocyte lineage cells^83^
Human *in vivo* findings	Activity-dependent EEG abnormalities^5, 34, 35^ a MRS-based GABA impairments^28^	Lower FA values on DTI^44, 45, 46, 47, 48, 49, 50, 51, 52, 53, 54, 55, 56, 57, 58^ a
Genetic support	CNVs^36^	GWAS common variants in myelin/oligodendrocyte gene sets^92, 93, 94, 95^ GWAS common variants enriched in mature oligodendrocytes^96^

Abbreviations: CNV, copy number variation; DTI, diffusion tensor imaging; EEG, electroencephalography; FA, fractional anisotropy; GABA, gamma-aminobutyric acid; GWAS, genome-wide association study; mRNA, messenger RNA; MRS, magnetic resonance spectroscopy; PV, parvalbumin.

a Present in first-episode, drug-naïve patients.

**Table 2:** Studies reporting myelination of GABAergic interneurons

*Study*	*Species*	*Brain region*	*Technique*	*Conclusion*
Somogyi *et al.*^100^	Cat	Visual cortex	Electron microscopy	Presence of single myelinated GAD+ cells
Mize *et al.*^118^	Cat	Superior colliculus	Electron microscopy	Presence of myelinated GABAergic neurons
Ong *et al.*^102^	Human	Frontal cortex	Electron microscopy	Presence of several myelinated GAT-1 axons
Ong *et al.*^102^	Monkey	Temporal cortex	Electron microscopy	Presence of several myelinated GAT-1 axons
Hendry *et al.*^106^	Monkey	Sensory-motor cortex	Electron microscopy	Presence of several myelinated layers III–V GABAergic neurons
DeFelipe *et al.*^107^	Monkey	Somatosensory cortex	Electron microscopy; [^3^H]GABA tracing	Presence of several myelinated GABAergic neurons
DeFelipe *et al.*^108^	Monkey	Sensorymotor cortex	Electron microscopy	Presence of several myelinated Layers III–V GABAergic neurons
Takasu *et al.*^109^	Monkey	Hypoglossal nucleus	Electron microscopy	Presence of several myelinated GABAergic neurons
Ralston *et al.*^110^	Monkey	Red nucleus	Electron microscopy	Presence of several myelinated GABAergic neurons
Jinno *et al.*^111^	Rat	Hippocampus	Single cell tracing; immunofluorescence	Presence of several myelinated GABAergic projection neurons
De Biasi *et al.*^113^	Rat	Thalamus	Electron microscopy	Presence of a few myelinated GABAergic axons
Conti *et al.*^114^	Rat	Cortex	Electron microscopy	Presence of several myelinated GAT-2 positive axons
Roberts *et al.*^121^	Rat	Inferior colliculus	Electron microscopy	Presence of several myelinated GABAergic neurons
Sawyer *et al.*^120^	Rat	Thalamus	Electron microscopy; light microscopy	Presence of several myelinated GABAergic neurons

Abbreviations: GABA, gamma-aminobutyric acid; GAD, glutamic acid decarboxylase; GAT, GABA transporter.

**Table 3:** Studies reporting myelination of parvalbumin-expressing interneurons

*Study*	*Species*	*Brain region*	*Technique*	*Conclusion*
Micheva *et al.*^125^	Mouse	Somatosensory cortex	Array tomography; electron microscopy; immunofluorescence	~25–50% of myelinated axons in the neocortex are GABAergic, of which nearly all are PV+
McGee *et al.*^97^	Mouse	Visual cortex	Immunofluorescence	~One-third of myelinated axons are PV+
Somogyi *et al.*^98^	Cat	Visual cortex	Electron microscopy	Presence of several myelinated basket cells
Somogyi *et al.*^99^	Cat	Visual cortex	Electron microscopy	Presence of two myelinated basket cells
Chung *et al.*^101^	Human	Frontal cortex	Immunofluorescence (CLARITY^a^)	Single figure of myelinated PV axons
Seress *et al.*^103^	Human	Hippocampus	Electron microscopy	Presence of a few myelinated PV axons
Hinova-Palova *et al.*^104^	Human	Claustrum	Electron microscopy; Immunofluorescence	Presence of several myelinated PV axons
Peters *et al.*^112^	Rat	Visual cortex	Electron microscopy	Presence of several myelinated basket cells
Wouterlood *et al.*^119^	Rat	Entorhinal cortex	Electron microscopy	Extensive presence of myelinated PV axons throughout all cortical layers
Gartner *et al.*^115, 116^;Brauer *et al.*^124^	Rat	Hippocampus	Immunofluorescence; electron microscopy	Majority of septohippocampal PV fibers show myelination, but not cholinergic ones
Kita *et al.*^123^	Rat	Neostriatum	Electron microscopy; light microscopy	Presence of several myelinated PV neurons
Freeman *et al.*^117^	Rat	Hippocampus *(in vitro)*	Hippocampal cultures; immunofluorescence	Myelination of PV neurons *in vitro*
Katsumaru *et al.*^122^	Rat	Hippocampus	Electron microscopy	Presence of myelinated PV neurons
Hu *et al.*^151^	Rat	Hippocampal dentate gyrus	Immunofluorescence	No myelinated PV fibers present

Abbreviations: GABA, gamma-aminobutyric acid; PV, parvalbumin.

a CLARITY (Chung *et al.*^100^) is a recently developed technique which enables immunofluorescence-based labeling and imaging of large volumes of structurally intact, optically transparent tissue.
